# Fabricating a Three-Dimensional Surface-Enhanced Raman Scattering Substrate Using Hydrogel-Loaded Freeze-Induced Silver Nanoparticle Aggregates for the Highly Sensitive Detection of Organic Pollutants in Seawater

**DOI:** 10.3390/s25082575

**Published:** 2025-04-18

**Authors:** Hai Liu, Yufeng Hu, Zhiyang Zhang

**Affiliations:** 1 School of Chemical Engineering, Ocean and Life Sciences, Dalian University of Technology, Panjin 124221, China; llhh@mail.dlut.edu.cn; 2 Coastal Zone Ecological Environment Monitoring Technology and Equipment Shandong Engineering Research Center, Shandong Key Laboratory of Coastal Environmental Processes, CAS Key Laboratory of Coastal Environmental Processes and Ecological Remediation, Yantai Institute of Coastal Zone Research, Chinese Academy of Sciences, Yantai 264003, China

**Keywords:** surface-enhanced Raman scattering (SERS), 3D SERS substrate, hydrogel, highly sensitive, uniformity, seawater detection

## Abstract

Surface-enhanced Raman scattering (SERS) spectroscopy faces challenges in achieving both high sensitivity and reproducibility for the detection of real samples, particularly in high-salinity matrices. In this study, we developed a high-performance, salt-resistant three-dimensional (3D) SERS substrate by integrating physically induced colloidal silver nanoparticle aggregates (AgNAs) with an agarose hydrogel. AgNAs were prepared using a freeze–thaw–ultrasonication method to minimize interference in SERS signals while significantly enhancing the detection efficiency of trace pollutants. The incorporation of the agarose hydrogel not only improved the substrate’s pollutant enrichment capability, but also effectively prevented the aggregation and sedimentation of AgNAs in salt solutions. The developed SERS substrate exhibited an ultralow detection limit of 10^−12^ M for Nile Blue (NB), with a 100-fold increase in sensitivity compared to colloidal AgNAs and drop-cast AgNAs solid substrates. The analytical enhancement factor (AEF) for malachite green (MG) achieved a value of 1.4 × 10^7^. Furthermore, the substrate demonstrated excellent signal uniformity, with a relative standard deviation (RSD) of 6.74% within a 200 μm × 200 μm detection area and also show a satisfactory RSD of only 9.38% within a larger area of 1 mm × 1 mm. Notably, the 3D SERS substrate exhibited excellent stability under high-salinity conditions (0.5 M NaCl) and successfully detected a model pollutant (MG) in real seawater samples using the standard addition method. This study provides a novel strategy for highly sensitive SERS detection of trace pollutants in saline environments, offering promising applications in environmental monitoring and marine pollution analysis.

## 1. Introduction

Surface-enhanced Raman scattering (SERS) spectroscopy has become a powerful tool for detecting trace targets due to its high sensitivity, rapid response, and non-destructive nature [1,2,3,4]. However, it is challenging to achieve satisfactory reproducibility and stability of SERS detection in real complex samples. Typically, there are two kinds of SERS substrates: solid SERS substrates and colloidal SERS substrates. Solid SERS substrates normally have two-dimensional (2D) structures, exhibiting higher stability in complex samples, compared with colloidal nanoparticle aggregates. However, for the preparation of uniform 2D SERS substrates, the current methods either suffer from a non-uniform distribution of nanoparticles (e.g., coffee ring effect) [5,6] using simple drop-casting methods, or require complicated steps (liquid/liquid nanoparticle assembly) and costly setups (e.g., physical deposition). Moreover, 2D SERS substrates inherently produce detection errors due to off-focus issues when using microscopic systems. In comparison, colloidal SERS substrates do not have these problems and usually demonstrate better reproducibility for the detection of individual samples. A colloidal SERS substrate in solution can be considered a three-dimensional (3D) SERS substrate, where nanoparticles are uniformly distributed in solution and their movement results in a uniform SERS signal.

To obtain satisfactory SERS sensitivity, it is necessary to prepare nanoparticle aggregates (nanoaggregates), which create strong electromagnetic hotspots between the particles thereby enhancing the sensitivity of colloidal SERS [7,8,9]. Common methods for preparing nanoaggregates include the use of chemical crosslinkers [10,11], surfactants [12], organic solvents [13], salt-induced aggregation, freeze-thaw cycles [14], and self-assembly techniques [15]. Among these, the recently developed freeze-thaw method is particularly favored due to its simplicity, high sensitivity, lack of chemical modifications, and preservation of the intrinsic surface characteristics (e.g., surface accessibility) of the nanoparticles [16]. However, despite the obvious advances of colloidal SERS substrates, all the colloidal nanoaggregates still face further aggregation and sedimentation in high-salinity environments and which significantly reduces their SERS stability [17,18].

In recent years, hydrogel-based SERS substrates have provided innovative solutions to the detection challenges in high-salinity environments [19,20,21]. The 3D network structure of hydrogels provides stable support for nanoparticles, effectively preventing their aggregation and sedimentation [22,23]. Additionally, the properties of the hydrogel network can be tuned to precisely control the arrangement and spacing of nanoparticles, thereby optimizing the enhancement of SERS signals [24]. Hydrogels exhibit excellent hydrophilicity and high tunability, enabling them to maintain good stability under various environmental conditions. More importantly, the network structure of hydrogels can effectively adsorb target molecules and enhance their interaction with nanoparticles, thereby improving the detection capability for low-concentration pollutants [25,26,27].

**Scheme 1:**
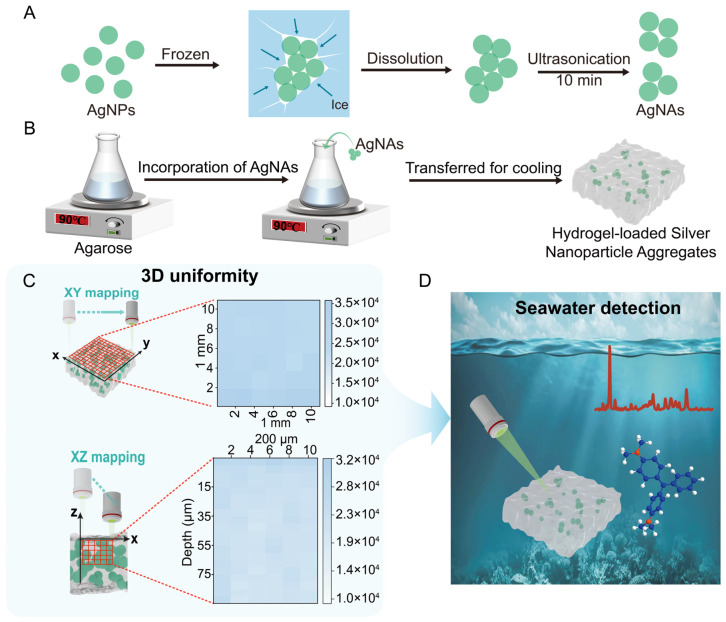
High SERS activity of 3D hydrogel-loaded silver nanoparticle aggregates and their application for detecting pollutants in seawater. (**A**) Schematic illustration of the preparation of Ag nanoparticle aggregates via a freeze-thaw-assisted ultrasound method; (**B**) Schematic illustration of the fabrication of a 3D hydrogel SERS substrate loaded with freeze-induced silver nanoparticle aggregates; (**C**) Schematic illustration of the three-dimensional uniformity of the 3D hydrogel SERS substrate; (**D**) Schematic illustration of the 3D hydrogel SERS substrate for marine pollutant detection.

In this study, we developed a novel 3D SERS substrate by integrating silver nanoparticle aggregates (AgNAs), synthesized via the freeze-thaw-assisted ultrasound method (Scheme 1A), into an agarose hydrogel matrix, thereby forming a hydrogel-loaded silver nanoparticle aggregates substrate (Scheme 1B). The introduction of the agarose hydrogel not only effectively immobilized the AgNAs, preventing their aggregation and sedimentation in high-salinity environments, but also enhanced the enrichment of target pollutants through its 3D network structure. The experimental results demonstrate that, compared to AgNA colloids and conventional solid substrates, the 3D hydrogel-loaded silver nanoparticle aggregates substrate exhibits a 100-fold increase in sensitivity for detecting the model molecule Nile Blue (NB), achieving an ultralow detection limit of 10^−12^ M. And the substrate exhibits excellent signal uniformity (with a relative standard deviation (RSD) of 6.74% within a 200 μm × 200 μm detection area. The 3D SERS substrate even shows good uniformity with the RSD of only 9.38% within a large area of 1 mm × 1 mm and a signal retention of 78% over a depth of 100 μm along the laser direction) (Scheme 1C). Moreover, the developed substrate maintains stable pollutant detection capability under high-salinity conditions (0.5 M NaCl) and exhibits an excellent performance in real seawater samples (Scheme 1D), further demonstrating its practical application potential in complex aquatic environments. The innovation of this study lies in the integration of freeze–thaw ultrasonically synthesized AgNAs with agarose hydrogel, resulting in the successful development of a high-performance, salt-tolerant SERS substrate. This strategy provides a new approach for the detection of trace pollutants in high-salinity water samples, offering broad application prospects in environmental monitoring, marine pollution analysis, and related fields.

## 2. Materials and Methods

### 2.1. Chemicals

Silver nitrate (AgNO_3_), glycerol, sodium citrate, and sodium chloride (NaCl) were purchased from Sinopharm Chemical Reagent Co., Ltd., Shanghai, China; agarose, crystal violet (CV), nile blue A (NB), methylene blue (MB) and malachite green (MG) were purchased from Shanghai Macklin Biochemical Technology Co., Ltd. Shanghai, China; glass capillary tubes (inner diameter: 0.9–1.1 mm) were produced by West China Medical University Instrument Factory, Chengdu, China.

### 2.2. Instruments

The UV/Vis/NIR absorption spectra were recorded on a Thermo Scientific NanoDrop 2000/2000C spectrophotometer (Thermo Scientific, Waltham, MA, USA). SERS was measured using a Renishaw in Via Raman microscope (Thermo Scientific, Waltham, MA, USA). The 633 nm lasers were focused by a 5× microscope objective for the sample solution. Scanning electron microscopy (SEM) images were obtained on an S-4800 field emission scanning electron microscope (Hitachi, Tokyo, Japan).

### 2.3. Preparation of AgNAs

Monodisperse silver nanoparticles (AgNPs) were synthesized following a previously reported method [28,29]. Specifically, deionized water (250 mL) containing glycerol (1 mL) was heated to 95 °C, followed by the addition of silver nitrate (45 mg) and sodium citrate (5 mL, 1%) under vigorous stirring. After continuous heating for 30 min, the solution gradually turned greenish brown, indicating the successful formation of monodisperse AgNPs. The reaction mixture was then cooled to room temperature and stored at 4 °C. The synthesized AgNPs were subsequently concentrated tenfold and subjected to a freeze-thaw process by freezing at −20 °C for 12 h, followed by thawing at room temperature. Finally, the thawed dispersion was sonicated for 10 min to obtain AgNAs.

### 2.4. Synthesis of 3D Hydrogel-Loaded Silver Nanoparticle Aggregates

In a total reaction system of 2 mL, 1 mL of 2% agarose solution was mixed with 50 μL (0.5:1), 75 μL (0.75:1), 100 μL (1:1), 200 μL (2:1), and 300 μL (3:1) of a 10-fold-concentrated AgNAs solution, with the remaining volume supplemented with deionized water. The mixture was heated to 90 °C under continuous stirring until homogenized, then rapidly transferred into a Petri dish and allowed to cool at room temperature to form 3D hydrogel-loaded silver nanoparticle aggregates with different AgNA loadings.

### 2.5. Spike Detection of MG in Seawater

Seawater samples were collected from the Yantai coastal area, with sampling points set at 500 m intervals, resulting in a total of four sampling points. All samples were pretreated using a 0.45 μm microporous filter to remove suspended particles. To establish a quantitative analysis model, one sample was selected for a spiking experiment, with malachite green standard solutions added to achieve final concentrations of 5 × 10^−7^ M, 1 × 10^−7^ M, 5 × 10^−8^ M, 1 × 10^−8^ M, and 5 × 10^−9^ M. Raman spectroscopy was used to measure the intensity of the characteristic peak at 1619 cm^−1^, and a linear regression model was established between malachite green concentration and Raman signal intensity. Subsequently, the remaining three environmental samples were subjected to method validation, with each sample spiked with 5 × 10^−8^ M of malachite green standard solution, and Raman spectroscopy analysis was performed under the same conditions.

## 3. Results

### 3.1. Preparation and Characterization of Hydrogel-Loaded 3D SERS Substrate

**Figure 1:**
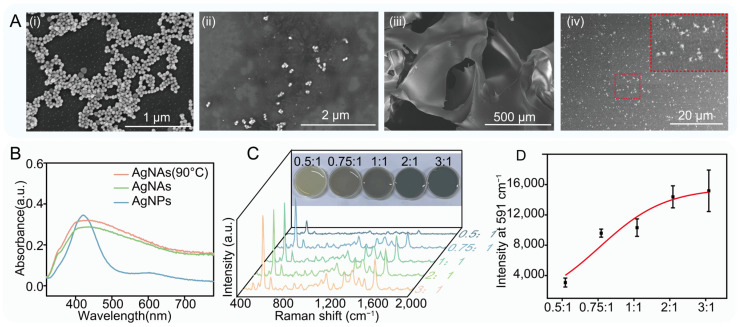
(**A**) SEM images of AgNPs (i), AgNAs (ii), and 3D hydrogel-loaded silver nanoparticle aggregates (iii,iv). (**B**) UV-Vis spectra of AgNPs and AgNAs. (**C**) Photographs of 3D hydrogel SERS substrates with varying AgNAs contents, along with the Raman spectra of NB molecules detected; (**D**) statistical plot of the Raman signal intensity of NB at 591 cm^−1^ on the substrate.

Based on the previous reports, silver nanoparticles (AgNPs) were synthesized using a citrate stabilization method. Subsequently, colloidal silver nanoparticle aggregates (AgNAs) with controlled sizes were prepared via a freeze–thaw–ultrasonication strategy developed by our group [16]. The AgNAs were then incorporated into an agarose hydrogel to fabricate a 3D hydrogel-loaded AgNA substrate. Figure 1A presents the scanning electron microscopy (SEM) images of different materials, including AgNPs (i), AgNAs (ii), and the 3D hydrogel-loaded AgNA substrate (iii–iv). As observed in the images, the synthesized AgNPs exhibit a uniform spherical morphology with a narrow size distribution. After the freeze–thaw–ultrasonication treatment, the monodisperse AgNPs formed aggregated structures. Within the hydrogel network, the AgNAs maintained their aggregated state without significant dispersion or structural disruption.

Figure 1B presents the UV-Vis absorption spectra of AgNPs and AgNAs. The monodisperse AgNPs exhibited a brown–green color (Figure S1) with a characteristic absorption peak around 420 nm. In contrast, AgNAs formed via the freeze–thaw–ultrasonication process appeared dark brown (Figure S1) and displayed a distinct aggregation-induced absorption peak in the spectrum [16], confirming the formation of nanoaggregates. Furthermore, after heating at 90 °C for 1 min, neither the color (Figure S1) nor the absorption spectrum of AgNAs showed significant changes, indicating that the aggregation state remained stable during the mixing and heating process with the agarose hydrogel.

Using Nile Blue (NB) as a probe molecule, the SERS performance of hydrogel-loaded Ag nanoparticle aggregates with varying Ag contents was characterized. As shown in Figure 1C, with an increase in AgNAs content, the color of the hydrogel gradually shifts from transparent to dark brown, accompanied by a significant enhancement in the Raman signal of NB molecules. Statistical analysis of the Raman peak intensity at 591 cm^−1^ (Figure 1D) indicates that as the AgNAs content increases from 0.5:1 to 3:1, the Raman signal intensity increases from 3077 to 15,079 cps. When the AgNAs content exceeds 2:1, the signal intensity increase plateaus, suggesting that the contribution of Ag content to the SERS enhancement effect has reached saturation. Additionally, the spatial uniformity of the SERS substrate was evaluated by calculating the relative standard deviation (RSD) of different sampling points (Figure S2). Taking into account both the SERS enhancement performance (Raman signal intensity) and spatial uniformity (RSD value), we selected the SERS hydrogel substrate with a 2:1 AgNAs content as the optimal condition. All subsequent experiments were conducted using this optimized SERS substrate.

### 3.2. High Sensitivity of Hydrogel-Loaded 3D SERS Substrate

**Figure 2:**
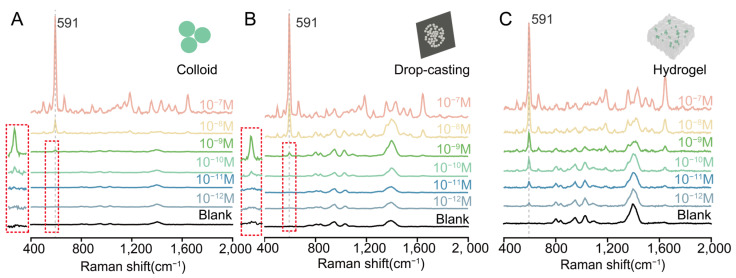
Background signals and SERS spectra of NB solutions (1 × 10^−7^ M to 1 × 10^−12^ M) for Ag aggregate colloid (**A**), droplet-based AgNAs solid substrate (**B**), and 3D hydrogel-loaded silver nanoparticle aggregate substrate (**C**).

The nanoaggregate structure forms strong electromagnetic hotspots between nanoparticles, significantly enhancing Raman signals, while the hydrogel further improves the SERS detection performance through enrichment effects [30,31]. In this study, Ag aggregates, droplet-cast AgNAs solid substrates, and 3D hydrogel-loaded silver nanoparticle aggregates were employed as SERS substrates. Stepwise dilution experiments of Nile Blue (NB) were conducted to determine the lowest concentration that generated a distinguishable SERS signal on each substrate. This approach was used to evaluate the detection limits and SERS enhancement performance of the different substrates. The results show that the detection limits for NB on Ag aggregates (Figure 2A) and AgNAs solid substrates (Figure 2B) are both 10^−10^ M, while the detection limit for the 3D hydrogel-loaded silver nanoparticle aggregates is improved to 10^−12^ M (Figure 2C). Compared with the previously reported studies (Table S1), the proposed method exhibits superior detection sensitivity.

This indicates that combining aggregates with hydrogel significantly enhances SERS sensitivity. To further demonstrate the enhancement performance of the 3D hydrogel-supported silver nanoparticle aggregates, crystal violet (CV), and malachite green (MG) were employed as probe molecules. The corresponding SERS analytical enhancement factors (AEFs) were calculated to be 1.2 × 10^7^ (Figure S3) and 1.4 × 10^7^ (Figure S4), further confirming the excellent SERS enhancement capability of the substrate.

### 3.3. Spatial Uniformty of Hydrogel-Loaded 3D SERS Substrate

**Figure 3:**
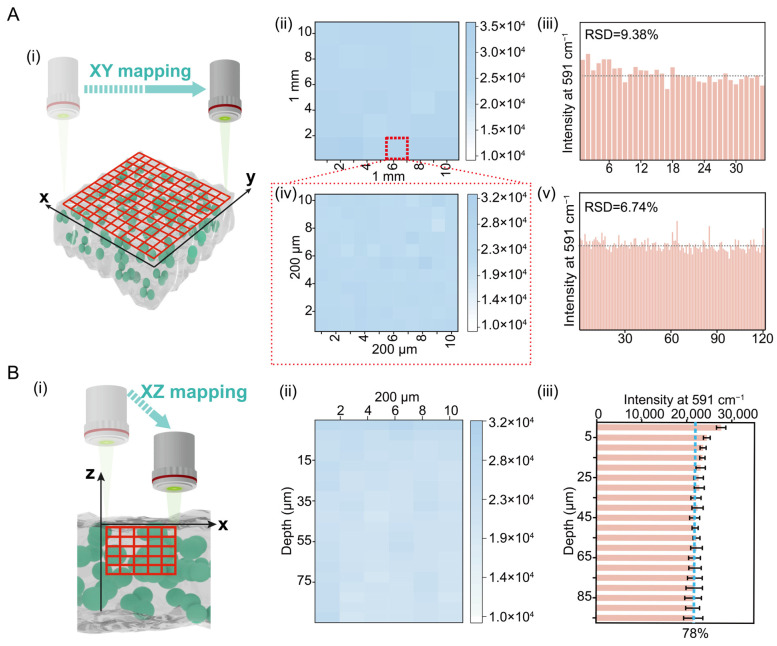
(**A**) (i) Schematic of the SERS mapping of 3D hydrogel-loaded silver nanoparticle aggregates perpendicular to the laser direction; (ii) mapping of hotspot images and (iii) statistical analysis of the Raman signal intensity at 591 cm^−1^ across a 1 mm × 1 mm area perpendicular to the laser direction; (iv) mapping of hotspot images and (v) statistical analysis of the Raman signal intensity at 591 cm^−1^ across a 200 μm × 200 μm area perpendicular to the laser direction. (**B**) (i) Schematic of the SERS mapping of 3D hydrogel-loaded silver nanoparticle aggregates parallel to the laser direction; (ii) mapping hotspot image in a 200 μm × 100 μm area parallel to the laser direction; (iii) statistical analysis of the variation in the intensity of the characteristic peak at 591 cm^−1^ with depth.

In practical applications, SERS substrates must provide stable and uniform signal enhancement to ensure the reliability and reproducibility of the detection results [32]. To evaluate the spatial uniformity of the 3D hydrogel-loaded silver nanoparticle aggregates (AgNAs) substrate, this study employs spatially resolved SERS mapping to quantitatively characterize its performance. The Raman signal intensity at different sampling points and their relative standard deviation (RSD) were statistically analyzed. Figure 3A(i) shows a schematic of the SERS mapping of the 3D hydrogel-loaded silver nanoparticle aggregates substrate in the direction perpendicular to the laser. SERS spectra were recorded in areas of 1000 μm × 1000 μm (1 mm × 1mm) and 200 μm × 200 μm with step sizes of 200 μm and 20 μm, respectively (Figure S4(i,ii)). The SERS mapping results, as shown in Figure 3A(ii–v), indicate that the RSD of the Raman signal intensity within the 200 μm × 200 μm area is 6.74%, and the RSD within the 1000 μm × 1000 μm area is 9.38%. In contrast, the AgNAs solid substrate exhibits poorer macro-morphology and signal uniformity under Raman microscopy, with an RSD of 25.48% (Figure S5). These results demonstrate that the 3D hydrogel-loaded silver nanoparticle aggregates substrate exhibits better signal uniformity in the direction perpendicular to the laser.

Figure 3B(i) presents a schematic illustration of the SERS mapping of the 3D hydrogel-loaded silver nanoparticle aggregate substrate along the direction parallel to the laser. A depth scan was performed within a 200 μm × 100 μm SERS region using a planar step size of 40 μm and a depth step size of 5 μm (Raman spectra are shown in Figure S4(iii)). As shown in Figure 3B(ii), the SERS mapping results were obtained, and the Raman intensity at 591 cm^−1^ for spectra collected at the same depth was statistically analyzed, as illustrated in Figure 3B(iii). Even at a depth of 100 μm, the signal intensity remained as high as 78%. Notably, within the depth range of 30 to 100 μm, the Raman signal exhibited minimal attenuation. These findings demonstrate that the 3D hydrogel-loaded silver nanoparticle aggregates exhibit excellent uniformity along the direction parallel to the laser.

The statistical analysis of the Raman characteristic peak intensity at 591 cm^−1^ for three independently prepared batches of 3D hydrogel-loaded silver nanoparticle aggregates revealed a relative standard deviation (RSD) of only 7.8% (Figure S7), indicating the excellent inter-batch reproducibility of the fabricated SERS substrate. This result strongly supports the stability and reliability of the preparation process developed in this study, confirming its suitability for practical applications. Furthermore, after storing the substrate in deionized water for 24 h, it retained more than 94% of the initial signal intensity, further demonstrating its good short-term stability (Figure S8).

### 3.4. Stability of Hydrogel-Loaded 3D SERS Substrate Under High-Salt Conditions

The salinity of water has a significant impact on the dispersion, aggregation state, and adsorption behavior of nanoparticles and target molecules. High salinity can lead to excessive aggregation or the precipitation of nanoparticles, thereby reducing the sensitivity and reliability of detection [33]. This study investigates the effect of salinity on the detection performance of Ag aggregates and their 3D hydrogel-loaded composite substrates. Ag aggregates and 3D hydrogel-loaded silver nanoparticle aggregates were incubated with pure NB solution and NB solution containing 0.5 M NaCl for 10 min, followed by detection every 5 min up to 30 min to evaluate the impact of salinity on their performance.

**Figure 4:**
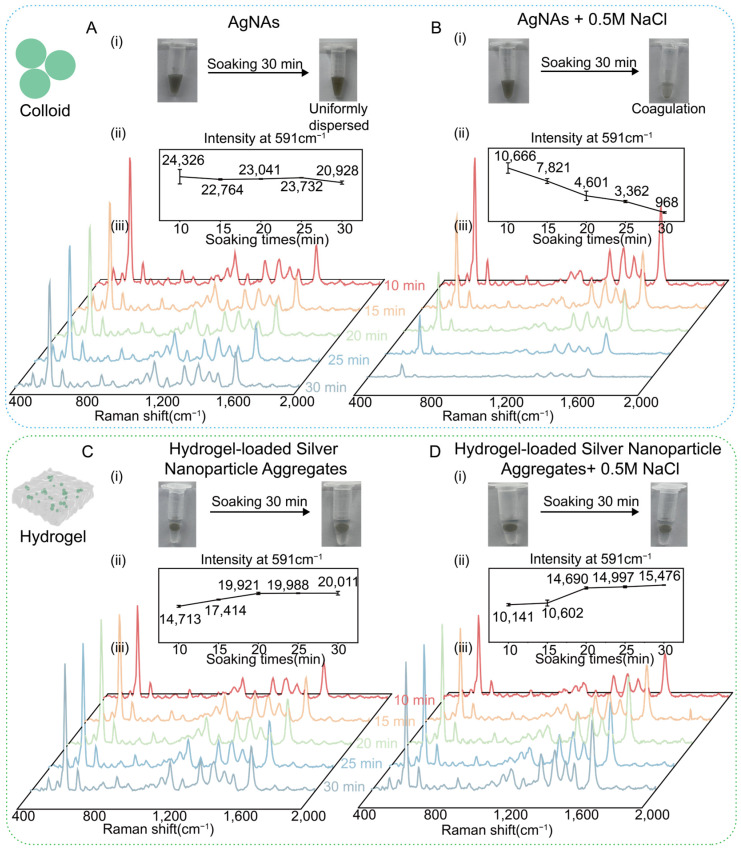
(**A**) AgNAs in 10^−7^ NB solution: (i) Photographs of the dispersion state over 30 min; (ii) statistical analysis of the NB characteristic peak intensity at 591 cm^−1^ over time (10–30 min); (iii) Raman spectra at different time points. (**B**) AgNAs in 10^−7^ M NB solution containing 0.5 M NaCl: (i) photographs of the dispersion state over 30 min; (ii) statistical analysis of the NB characteristic peak intensity at 591 cm^−1^ over time (10–30 min); (iii) Raman spectra at different time points. (**C**) Three-dimensional hydrogel-loaded silver nanoparticle aggregates in 10^−7^ M NB solution: (i) photographs of the state changes over 30 min; (ii) statistical analysis of the NB characteristic peak intensity at 591 cm^−1^ over time (10–30 min); (iii) Raman spectra at different time points. (**D**) Three-dimensional hydrogel-loaded silver nanoparticle aggregates in 10^−7^ M NB solution containing 0.5 M NaCl: (i) photographs of the state changes over 30 min; (ii) statistical analysis of the NB characteristic peak intensity at 591 cm^−1^ over time (10-30 min); (iii) Raman spectra at different time points.

In pure water, Ag aggregates maintained good dispersion over the 30 min period (Figure 4A(i)), whereas in the 0.5 M NaCl solution, significant sedimentation of Ag aggregates occurred over the same period (Figure 4B(i)). Raman signal detection of the NB solution was then used to further assess the impact of salinity on the SERS performance of Ag aggregates. Panels (ii) and (iii) in Figure 4A,B show the Raman spectra and statistical analysis of the 591 cm^−1^ characteristic peak intensity of Ag aggregates from 10 to 30 min in pure water and 0.5 M NaCl solution. The results show that in pure water, the Raman signal intensity of NB remains stable, with the intensity of the 591 cm^−1^ characteristic peak being 24,326 cps at 10 min and 20,928 cps at 30 min. In contrast, in the 0.5 M NaCl solution, the 591 cm^−1^ peak intensity significantly decreased from 10,666 cps at 10 min to 968 cps at 30 min. This indicates that salinity significantly weakened the detection ability of Ag aggregates for pollutants, limiting their application in real-water monitoring.

Figure 4C,D show the effect of salinity on the detection performance of the 3D hydrogel-loaded silver nanoparticle aggregates substrate in the NB solution. Panel (i) in Figure 4C,D compares the macroscopic morphology of the 3D hydrogel-loaded silver nanoparticle aggregates substrate before and after 30 min of immersion in pure water and 0.5 M NaCl solution. The results demonstrate that, regardless of the solution type, the Ag aggregate-hydrogel composite substrate retains a stable morphology and does not disperse over 30 min. Panels (ii) and (iii) in Figure 4C,D display the Raman spectra and statistical analysis of the 591 cm^−1^ peak intensity of the Ag aggregate–hydrogel composite substrate in NB solution in pure water. The results show that the SERS signal gradually increases between 10 and 20 min, with the 591 cm^−1^ peak intensity rising from 14,713 at 10 min to 19,921 at 20 min, and the signal intensity remains stable from 20 to 30 min, suggesting that adsorption reachs saturation after 20 min. Figure 4D displays the Raman spectra and 591 cm^−1^ peak intensity statistics for the Ag aggregate–hydrogel composite substrate in the NB solution with 0.5 M NaCl. The results indicate that the 591 cm^−1^ peak intensity increases from 10,141 cps at 10 min to 14,690 at 20 min, and the signal intensity stabilizes from 20 to 30 min, indicating that adsorption approaches saturation. Compared to pure water, the Raman signal intensity in the 0.5 M NaCl solution was lower, likely due to the effect of salinity on the hydrogel’s adsorption capacity for NB; the 3D hydrogel-loaded silver nanoparticle aggregate substrate was still able to stably detect pollutant signals in high salinity conditions, effectively overcoming the challenges posed by salinity on Ag aggregates. 

### 3.5. Detection of Marine Pollutants Using Hydrogel-Loaded 3D SERS Substrate

**Figure 5:**
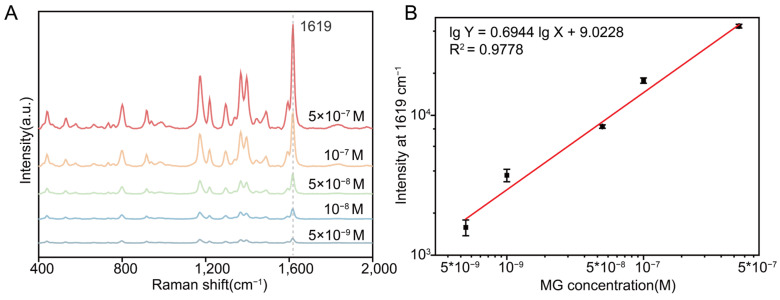
(**A**) Raman spectra of MG in seawater. (**B**) The linear relationship between the signal intensity of the characteristic peak of MG at 591 cm^−1^ and its concentration in seawater.

To evaluate the feasibility and practicality of the fabricated 3D hydrogel-loaded silver nanoparticle aggregates substrate for pollutant detection in the real seawater matrix, malachite green (MG), as a typical antibacterial agent for aquaculture, was chosen as the model pollutant in this work. The different samples were prepared by spiking varying concentrations in the seawater without MG and the SERS spectra were measured using the hydrogel-loaded 3D SERS substrate (Figure 5A). Quantitative analysis was performed by correlating the intensity of the characteristic peak at 1619 cm^−1^ (*Y*) with the MG concentration (*X*), and a linear regression equation was established (Figure 5B). The regression equation was of the following form:

lg Y=0.6944 lg X+9.0228

Further validation of the method’s accuracy was carried out through spiked recovery experiments, with recovery rates of 90.4%, 95.4%, and 108.4% observed for seawater samples from three different sites (Table 1).

Moreover, the fabricated 3D SERS substrate was able to reliably identify the characteristic Raman signals of various organic pollutants in seawater, including methylene blue, CV, and NB (Figure S9), further demonstrating its applicability and reliability for pollutant detection in complex marine environments. The experimental results indicate that the hydrogel-loaded silver nanoparticle aggregates (AgNAs) SERS substrate maintains an excellent detection performance in complex seawater matrices, highlighting its strong potential for practical applications in environmental monitoring.

## 4. Conclusions

This study presents a 3D SERS substrate by combining freeze-prepared silver nanoparticle aggregates with agarose hydrogel with high sensitivity and salt-resistance. The SERS substrate effectively harnesses the strong electromagnetic hotspots and the intrinsic surface accessibility of freeze-prepared silver nanoparticle aggregates, as well as molecular enrichment and stable structure of the 3D hydrogel network structure. This combination significantly enhances the sensitivity, signal uniformity, and stability of the SERS substrate in high-salinity water (0.5 M NaCl). The experimental results show that the 3D hydrogel-loaded silver nanoparticle aggregate substrate achieves a detection limit for NB as low as 10^−12^ M, which is superior to AgNA colloidal and solid substrates with detection limits of 10^−10^ M. Moreover, the substrate demonstrates excellent signal uniformity, with an RSD of 9.38% over a 1 mm × 1 mm area perpendicular to the laser direction. Even in high-salinity seawater, the substrate remains stable in detecting environmental pollutants. These advantages make the 3D hydrogel-loaded silver nanoparticle aggregate substrate a promising tool for environmental monitoring, marine pollution analysis, and pollutant detection in related fields.

## Figures and Tables

**Table 1:** Detection of MG in seawater samples by the hydrogel-loaded 3D SERS substrate.

Sample	Spiked (nM)	Detected (nM)	Recovery (%)
1	50	45.2	90.4
2	50	47.7	95.4
3	50	54.4	108.4

## Data Availability

Data are available upon request from the corresponding authors.

## Supplementary Materials

The following supporting information can be downloaded at: https://www.mdpi.com/article/10.3390/s25082575/s1, Figure S1: Photographs of monodisperse Ag nanoparticles, AgNAs, and AgNAs (90 °C) colloids; Figure S2: Raman signals of NB on the SERS substrate with different AgNAs loading amounts, as well as the average Raman intensity and relative standard deviation of the characteristic peak at 591 cm^−1^; Figure S3: Calculation of the AEF for CV using 3D SERS substrates; Figure S4: Calculation of the AEF for MG using 3D SERS substrates; Figure S5: Raman spectrum of the 3D uniformity test of the substrate; Figure S6: Microscopic images of the 3D hydrogel-loaded silver nanoparticle aggregates, the silver nanoparticle aggregate solid substrate, and uniformity analysis; Figure S7: Batch uniformity images of the 3D hydrogel-loaded silver nanoparticle aggregate substrate; Figure S8: Short-term stability performance of 3D SERS substrates; Figure S9: Raman spectra of MB, CV, and NB in seawater; Table S1. Comparison of detection limits for NB in recent SERS-based studies. References [16,34,35,36,37,38,39] are cited in the supplementary materials.
