# Supplementary material for: Fabricating a Three-Dimensional Surface-Enhanced Raman Scattering Substrate Using Hydrogel-Loaded Freeze-Induced Silver Nanoparticle Aggregates for the Highly Sensitive Detection of Organic Pollutants in Seawater
Source: Sensors (Basel). 2025 Apr 18;25(8):2575. doi: 10.3390/s25082575 (PMC12031476; doi:10.3390/s25082575)
Supplement: Supplementary file 1 [file sensors-25-02575-s001.zip › sensors-3579189-supplementary.pdf]

Supporting Information for

Fabricating a Three-Dimensional Surface-Enhanced Raman Scattering Substrate Using Hydrogel-Loaded Freeze-Induced Silver Nanoparticle Aggregates for the Highly Sensitive Detection of Organic Pollutants in Seawater

Hai Liu [1], Yufeng Hu [1,*] and Zhiyang Zhang [2,*]

[1] School of Chemical Engineering, Ocean and Life Sciences, Dalian University of Technology, Panjin 124221, China; llhh@mail.dlut.edu.cn

[2] Coastal Zone Ecological Environment Monitoring Technology and Equipment Shandong Engineering Research Center, Shandong Key Laboratory of Coastal Environmental Processes, CAS Key Laboratory of Coastal Environmental Processes and Ecological Remediation, Yantai Institute of Coastal Zone Research, Chinese Academy of Sciences, Yantai 264003, China

* Correspondence: yufenghu@dlut.edu.cn (Y.H.); zyzhang@yic.ac.cn (Z.Z.)

1. Supporting figures

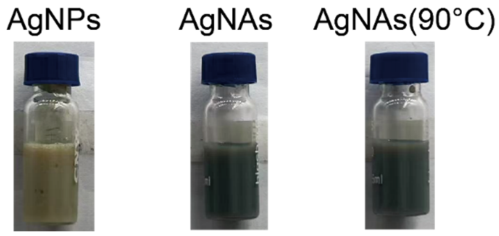

Figure S1. Photographs of monodisperse Ag nanoparticles, AgNAs, and AgNAs (90°C) colloids.

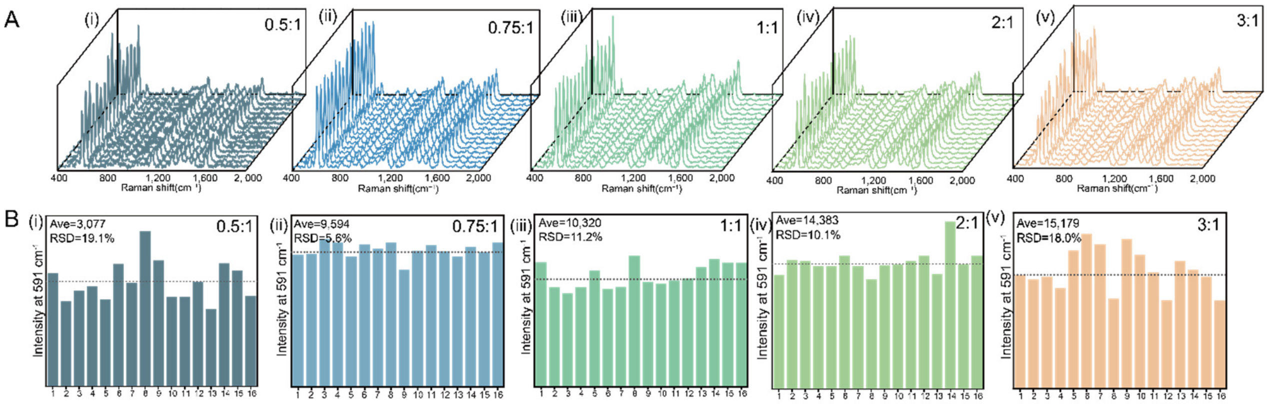

Figure S2. Raman signals of Nile Blue (NB) on the SERS substrate (A), along with the mean Raman intensity

and the relative standard deviation of the characteristic peak at 591 cm^{-1} (B). The mean Raman intensity and RSD for different Ag content substrates are as follows: the 0.5:1 substrate has a mean Raman intensity of 3,077 with an RSD of 19.1%; the 0.75:1 substrate has a mean intensity of 9,594 with an RSD of 5.6%; the 1:1 substrate has a mean intensity of 10,320 with an RSD of 11.2%; the 2:1 substrate has a mean intensity of 14,383 with an RSD of 10.1% (after removing the 14th outlier, the RSD decreases to 5.5%); and the 3:1 substrate has a mean intensity of 15,079 with an RSD of 18.0%.

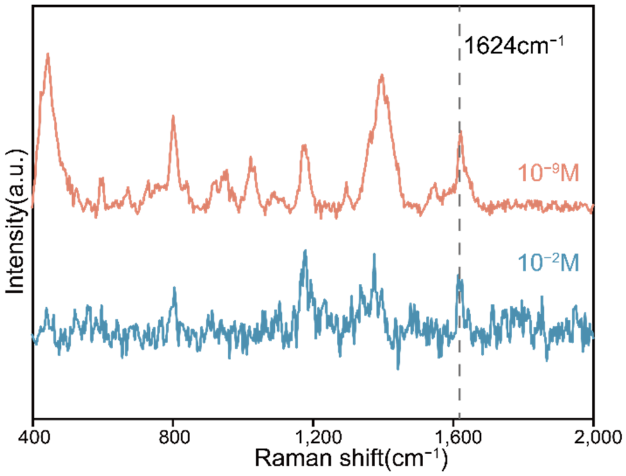

Figure S3. Raman spectrum of 10^{-2} M CV stock solution and surface-enhanced Raman spectrum of 10^{-9} M CV detected using the 3D hydrogel-loaded silver nanoparticle aggregates. The Raman characteristic peak intensity at 1624 cm^{-1} was 187.9 cps for conventional Raman spectroscopy and 216.9 cps for SERS. Based on the analytical enhancement factor (AEF) equation: $AEF = \frac{I_{SERS}/C_{SERS}}{I_{RS}/C_{RS}}$, the calculated AEF value was 1.2×10^{7}.

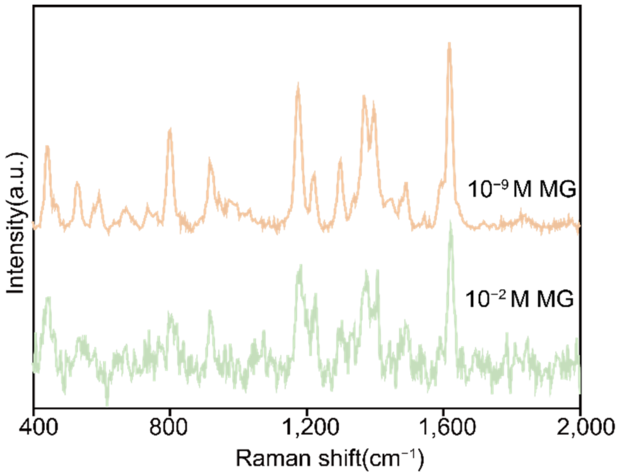

Figure S4. Raman spectrum of 10^{-2} M MG stock solution and surface-enhanced Raman spectrum of 10^{-9} M MG detected using the 3D hydrogel-loaded silver nanoparticle aggregates. The Raman characteristic peak intensity at 1619 cm^{-1} was 458.8 cps for conventional Raman spectroscopy and 623.1 cps for SERS. Based on the analytical enhancement factor (AEF) equation: $AEF = \frac{I_{SERS}/C_{SERS}}{I_{RS}/C_{RS}}$, the calculated AEF value was1.4×10^{7}.

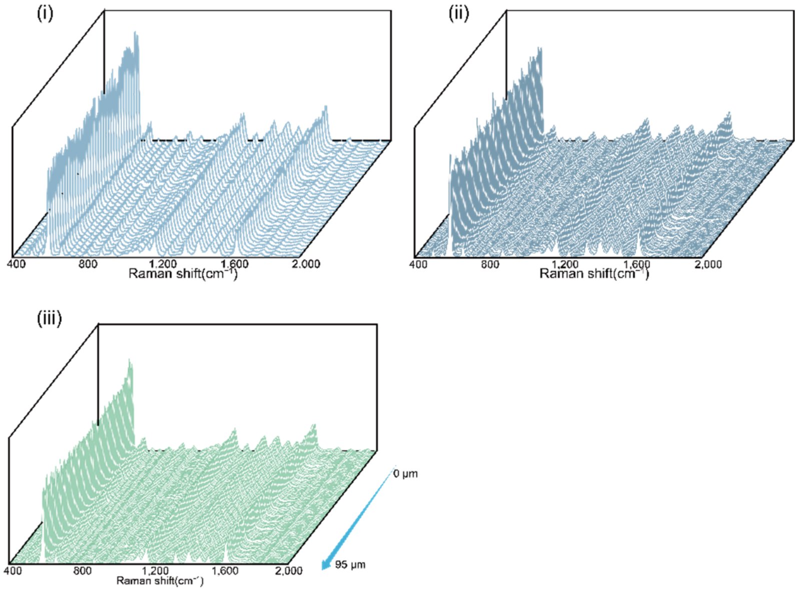

Figure S5. Raman spectra were recorded in SERS regions of 1,000 μm × 1,000 μm (i) and 200 μm × 200 μm (ii) perpendicular to the laser direction, and in SERS regions of 200 μm ×100μm (iii) parallel to the laser direction.

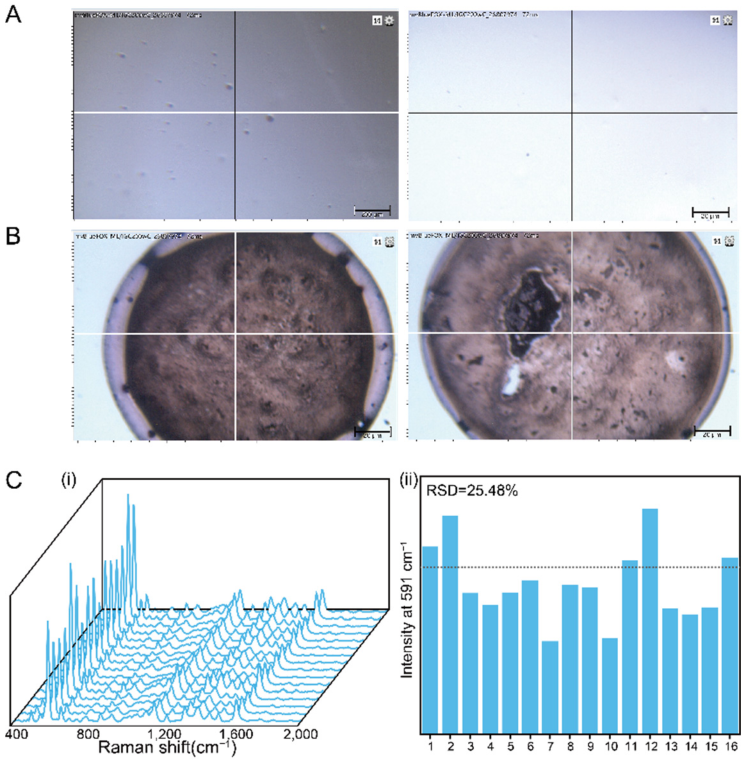

Figure S6. (A) Microscopic image of the 3D hydrogel-loaded silver nanoparticle aggregates; (B) Microscopic image of the silver nanoparticle aggregates drop cast; (C) Raman signals at different locations of NB on the AgNAs drop cast (i), bar chart of Raman characteristic peak (591 cm^{-1}) intensity at different locations of NB on the silver nanoparticle aggregates drop cast (ii).

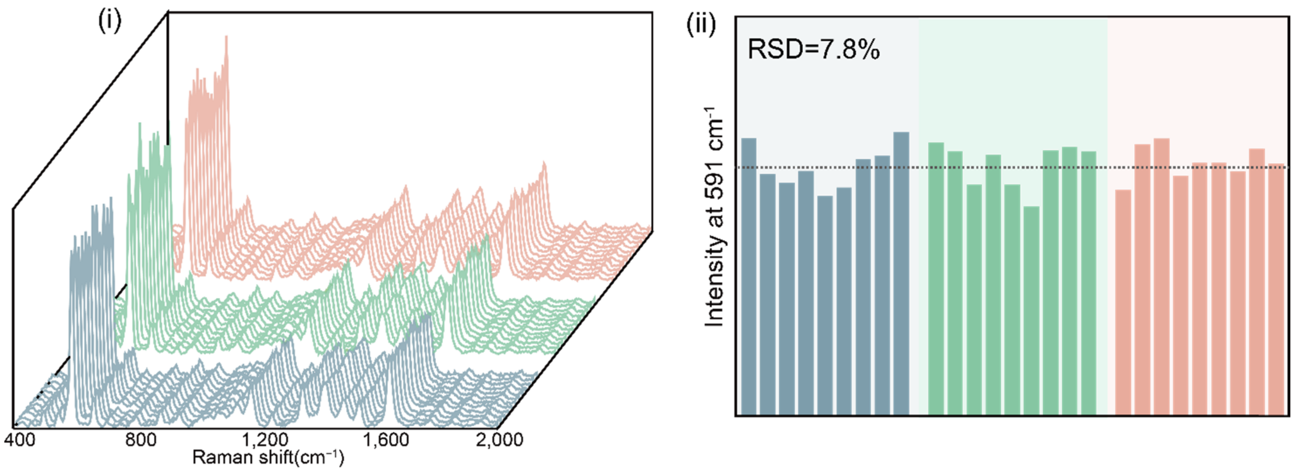

Figure S7. Mapping Raman signal images (i) and characteristic peak intensity statistics (ii) of three batches of 3D hydrogel-loaded silver nanoparticle aggregate substrates, measured perpendicular to the laser direction.

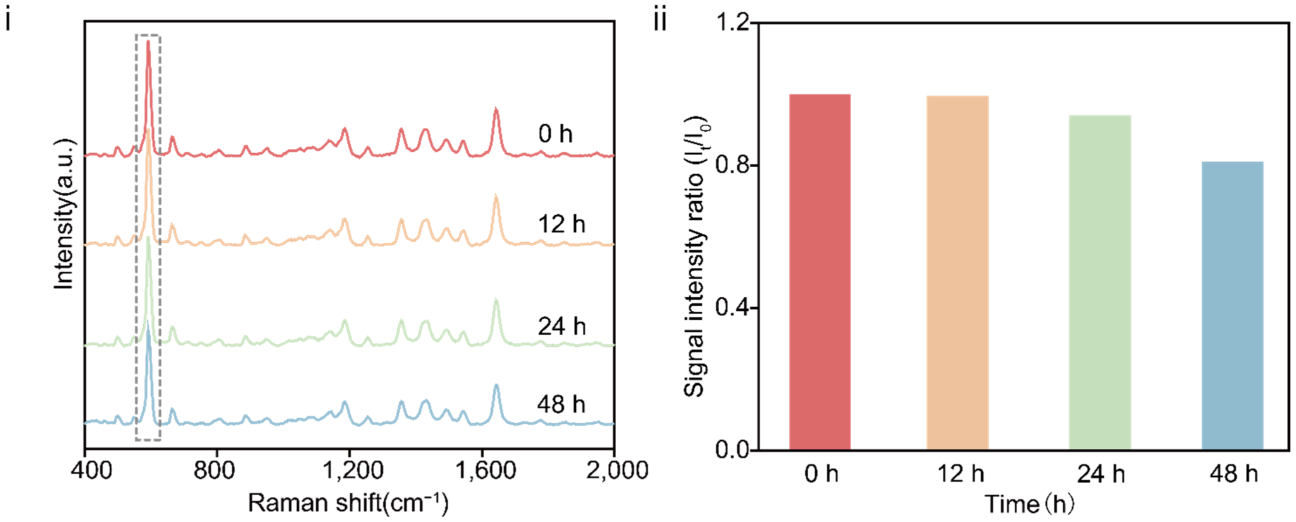

Figure S8 (i) the Raman signal of 10^{-7} M NB detected using the 3D SERS substrate over a 0 - 48h period, (ii) the ratio of the characteristic peak at 591 cm^{-1} relative to that at 0h at different time points.

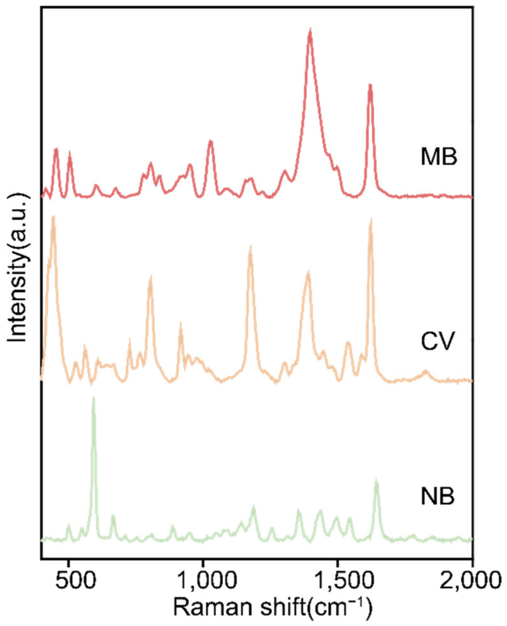

Figure S9 Raman spectra of 10^{-7} M Nile Blue (NB), Crystal Violet (CV), and Methylene Blue (MB) detected in seawater using the 3D SERS substrate.

Table S1. Comparison of Detection Limits for NB in Recent SERS-Based Studies

SERS Substrate	Detection Limit for NB	Reference
Graphene oxide wrapped Fe_3O_4@Au	1×10^{-10} M	[1]
AgNAs	3×10^{-9} M	[2]
Filter Paper -loaded Au NPs SERS substrate	1×10^{-9} M	[3]
Femtosecond laser patterned silicon embedded with gold nanostars	5×10^{-9} M	[4]
Ag@PS Composite	5×10^{-7} M	[5]
Mxenes-AuNP Hybrid Plasmonic 2D Microplates	1×10^{-10} M	[6]
Au nanostar-loaded laser-processed Ag substrates	5×10^{-11} M	[7]
3D SERS substrate	10^{-12} M	This work

References

1. Ding, G.; Xie, S.; Zhu, Y.; Liu, Y.; Wang, L.; Xu, F. Graphene oxide wrapped Fe3O4@Au nanohybrid as SERS substrate for aromatic dye detection. *Sensors and Actuators B: Chemical* **2015**, *221*, 1084-1093, doi:https://doi.org/10.1016/j.snb.2015.07.074.
2. Cheng, J.; Zhang, Z.; Zhang, L.; Miao, J.; Chen, Y.; Zhao, R.; Liu, M.; Chen, L.; Wang, X. Size-controllable colloidal Ag nano-aggregates with long-time SERS detection window for on-line high-throughput

detection. *Talanta* **2023**, *257*, 124358, doi:https://doi.org/10.1016/j.talanta.2023.124358.

3. Byram, C.; Rathod, J.; Moram, S.S.B.; Mangababu, A.; Soma, V.R. Picosecond Laser-Ablated Nanoparticles Loaded Filter Paper for SERS-Based Trace Detection of Thiram, 1,3,5-Trinitroperhydro-1,3,5-triazine (RDX), and Nile Blue. **2022**, *12*, 2150.
4. Moram, S.S.B.; Byram, C.; Soma, V.R. Femtosecond laser patterned silicon embedded with gold nanostars as a hybrid SERS substrate for pesticide detection. *RSC Advances* **2023**, *13*, 2620-2630, doi:10.1039/D2RA07859G.
5. Tian, X.; Yu, Q.; Kong, X.; Zhang, M. Preparation of Plasmonic Ag@PS Composite via Seed-Mediated In Situ Growth Method and Application in SERS. *Frontiers in chemistry* **2022**, *10*, 847203, doi:10.3389/fchem.2022.847203.
6. Chen, Z.; Liu, A.; Zhang, X.; Jiao, J.; Yuan, Y.; Huang, Y.; Yan, S. Mxenes–Au NP Hybrid Plasmonic 2D Microplates in Microfluidics for SERS Detection. **2022**, *12*, 505.
7. Rathod, J.; Byram, C.; Kanaka, R.K.; Sree Satya Bharati, M.; Banerjee, D.; Akkanaboina, M.; Soma, V.R. Hybrid Surface-Enhanced Raman Scattering Substrates for the Trace Detection of Ammonium Nitrate, Thiram, and Nile Blue. *ACS Omega* **2022**, *7*, 15969-15981, doi:10.1021/acsomega.2c01095.
